# Alteration of the gut microbiota in rhesus monkey with spontaneous osteoarthritis

**DOI:** 10.1186/s12866-021-02390-0

**Published:** 2021-11-27

**Authors:** Yaping Yan, Xiaoyan Yi, Yanchao Duan, Bin Jiang, Tianzhuang Huang, Briauna Marie Inglis, Bingrong Zheng, Wei Si

**Affiliations:** 1 School of Medicine, Yunnan University, Kunming, Yunnan China; 2 State Key Laboratory of Primate Biomedical Research, Institute of Primate Translational Medicine, Kunming University of Science and Technology, Kunming, China

**Keywords:** Spontaneous osteoarthritis, Rhesus macaque, Gut microbiota, Cartilage damage

## Abstract

**Background:**

The spontaneous osteoarthritis (OA) in rhesus macaque is similar to OA in human, which maintains an upright body posture and shows very similar biomechanical properties of bones to humans. At present, there is no good treatment for OA. This study aims to explore relationship between OA and intestinal microbiota, and provide a reference for the treatment of clinical OA.

**Results:**

We collected colonic contents of the 20 rhesus macaque (6–15 years old, female) for intestinal microbiota analysis by metagenomics sequencing, of which 10 were spontaneous OA monkeys and 10 were normal monkeys. Our results showed the diversity of gut microbiota in monkeys with OA was decreased compared to the normal monkeys (*p* = 0.16). Mollicutes, Tenericutes, *Coprobacillus* and *Faecalitalea* may be biomarkers for the monkeys of OA. *Lactobacillus* found significantly increased in OA monkeys. *Prevotella* and *Ruminococcus* were higher in the normal group than OA group. Zinc/manganese transport system permease protein (*p* = 0.0011) and Cyclopropane-fatty-acyl-phospholipid synthase (*p* = 0.0012) are a microbiota metabolic pathway related to cartilage production.

**Conclusions:**

Our results indicate that the diversity and composition of intestinal microbiota in monkeys with OA are different compared to the normal monkeys. we have found microbes that may be a biomarker for the diagnosis of osteoarthritis. Functional analysis of the microbiota also predicts cartilage damage in the monkeys with osteoarthritis. Non-human primates are closely related to humans, so this study can provide a reference for the development of drugs for the treatment of OA.

## Background

Osteoarthritis (OA) is the most common form of arthritis and the leading cause of chronic disability that mainly affects the knee and hip joints. The widespread existence of osteoarthritis has caused a heavy social and medical burden [1, 2]. Despite its great impact, the etiology and pathogenesis of OA remains blurry. Articular cartilage damages and osteophytes are the major features of OA. Currently, apart from the purpose of short-term pain relief, there is no efficient drug or therapy that is capable of effectively regenerating cartilage and curing the disease [3]. In addition, the anti-inflammatory drugs such as acetaminophen can inhibit the inflammation of osteoarthritis, but the side effects of these steroids and hormone drugs can cause severe addiction, endocrine disorders and obesity [4].

Many factors have been discovered to be associated with the occurrence of osteoarthritis including obesity, age, gender, genetic factors and so on [5]. The risk of osteoarthritis related to genetic factors seems to be relatively mild [6]. Non-genetic factors are the main cause of OA in humans and mice, which including obesity, aging, diet and gut microbiota [7–11]. Trillions of bacteria exist in the intestinal track [12]. The intestinal microbiome controls the expansion of pathogens and invasive microbiota, and maintains the integrity of the intestinal barriers [13]. The reduction of the beneficial microbiome can cause damage to the intestinal barrier. A study has shown that the high-fat diet given to obese mice led to obesity-related intestinal microbiome imbalance accompanied with the migration of macrophages to synovium of knee joints, which exacerbates the traumatic knee osteoarthritis [14]. Previous studies have found that the composition and diversity of microbiota in human osteoarthritis cartilage is different compared to that of healthy people [15]. Therefore, the close association between gut microbiome and osteoarthritis indicates that intestine microbes have the potential to be the biomarkers for diagnosis and can be targets for therapy of OA.

At present, there are very few studies on the intestinal microbes of OA. Animal models play an important role in drug and therapeutic development of OA [16]. Presently, rodents are the most commonly used animal models of OA, but are very different from humans in terms of biomechanical properties, structure of bones and body posture of joints and bones [17, 18]. Furthermore, Matrix metallopeptidase 1 (MMP-1) is a major collagenase related to human joint diseases that has been applies as a diagnostic marker for human osteoarthritis, but MMP-1 is not expressed in rodents [19]. Therefore, rodents are not suitable as animal models for OA research. Injecting collagenase into the joint cavities is the main way to generate OA model in rodents. However, the collagenase-induced rodent OA models show acute inflammation which directly leads to the destruction of cartilage, presenting an obvious difference of pathogenesis compared to human OA [16]. In contrast, the rhesus monkey maintains an upright body posture and shows very similar biomechanical properties of bones to humans [18], and typical spontaneous OA develops in adult rhesus monkeys with ageing [20]. Epidemiology and joint pathology have both shown that the rhesus monkey with spontaneous osteoarthritis are ideal animal models for human OA study [21]. Although it has been confirmed that the composition of microbes from cartilage are altered in patients with osteoarthritis, the cartilage microbes is not suitable for clinical diagnosis and therapeutic application because the sampling is invasive and not practical. Therefore, the present study aims to explore the relationship between OA and the gut microbes in the rhesus monkey with spontaneous osteoarthritis. We attempted to identify potentially biomarkers for diagnosis or auxiliary diagnosis of OA and provide valuable reference for microbiota therapy of human with osteoarthritis.

## Results

### Confirmation of spontaneous osteoarthritis models of rhesus macaque

**Fig. 1:**
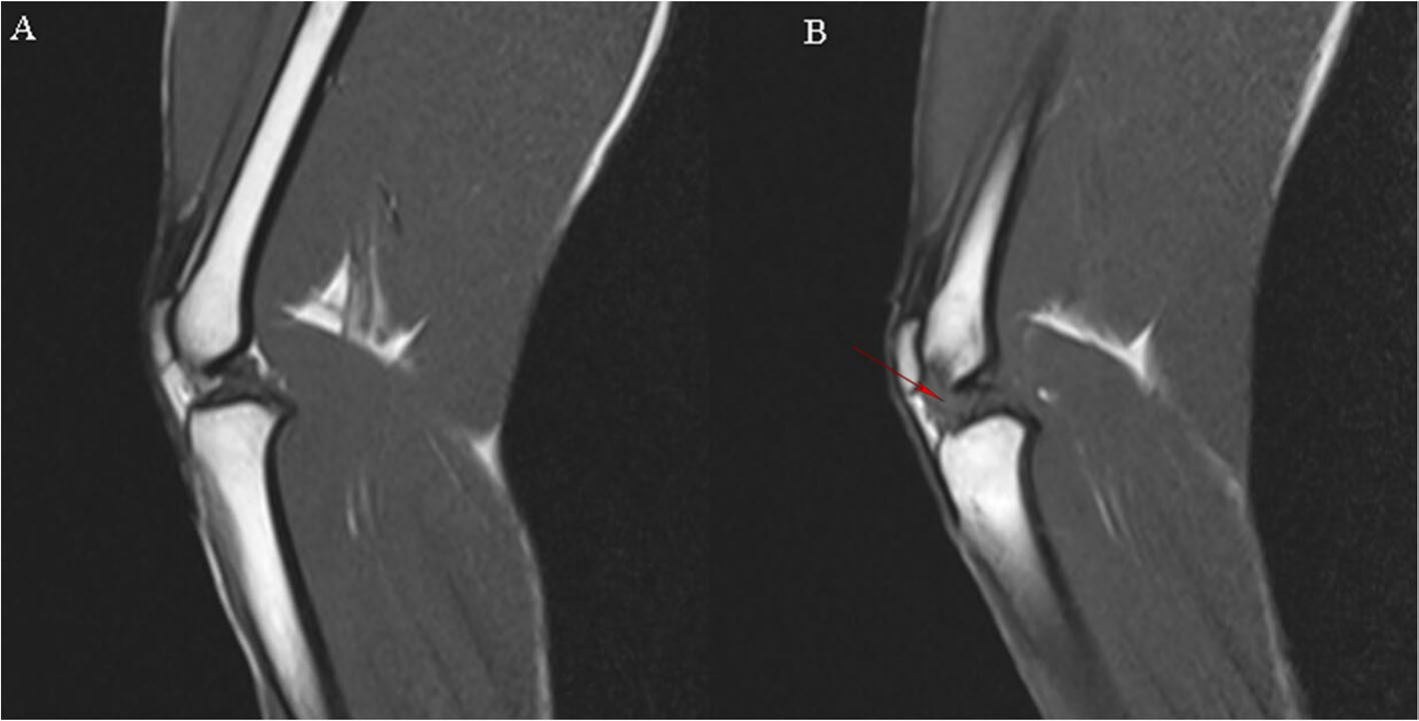
Rhesus monkey knee joints were analysis by MRI. **A** Picture of normal monkey knee. **B** Picture of osteoarthritis monkeys, the exudative patellar ligament and bone hyperplasia (red arrow) increased

The MRI image of the normal monkeys of knee joint shows a clear line of bone growth plate, smooth and boneless woven spine in cartilage, and smooth and even synovial tissue in the joint. In contrast, monkeys of osteoarthritis show significant joint tibia end damage and bone hyperplasia (Fig. 1).

### Microbiota diversity between OA and normal monkeys

**Fig. 2:**
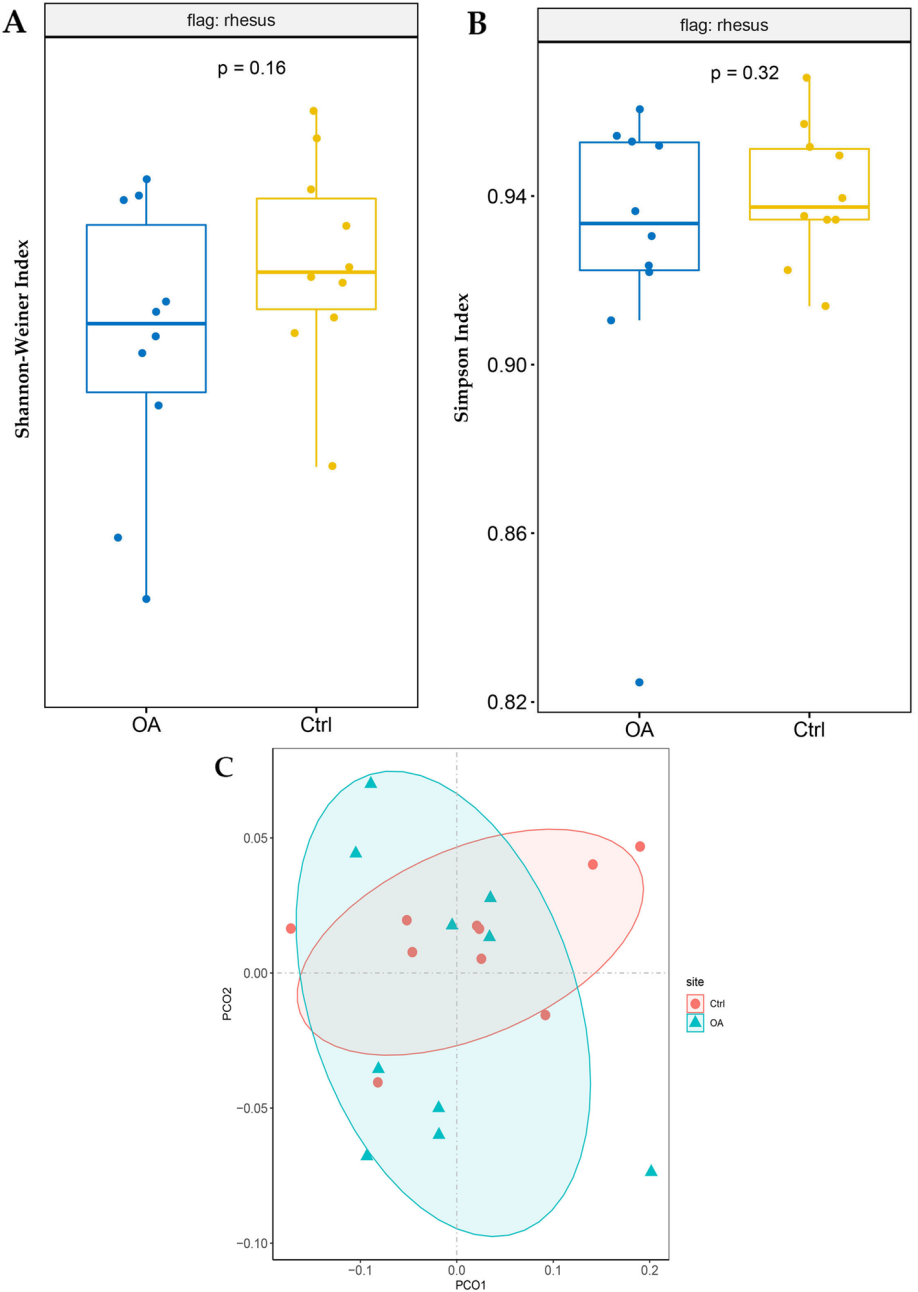
Analyze the alpha-diversity and beta-diversity indices of the gut microbes in the OA and normal monkeys. **A** Compare Shannon index based on OUT in the OA and normal monkeys. **B** Compare Simpson index based on the OTU counts in the OA and normal monkeys. Each box plot represents the median, interquartile range, minimum, and maximum values. **C** Unweighted PCA analysis in the OA and normal monkeys

Gut microbiota was characterized by metagenomics sequencing. Alpha diversity analysis including the Shannon and Simpson index showed that the fecal microbes of OA monkeys was less diverse compared to the normal monkeys (*p* = 0.16, *p* = 0.32). (Fig. 2A, Fig. 2B). Beta diversity by examining the unweighted Unifrac distance expounded that there is a separation between both groups (Fig. 2C).

### Shifty microbes may become potential biomarkers in OA diagnosis

**Fig. 3:**
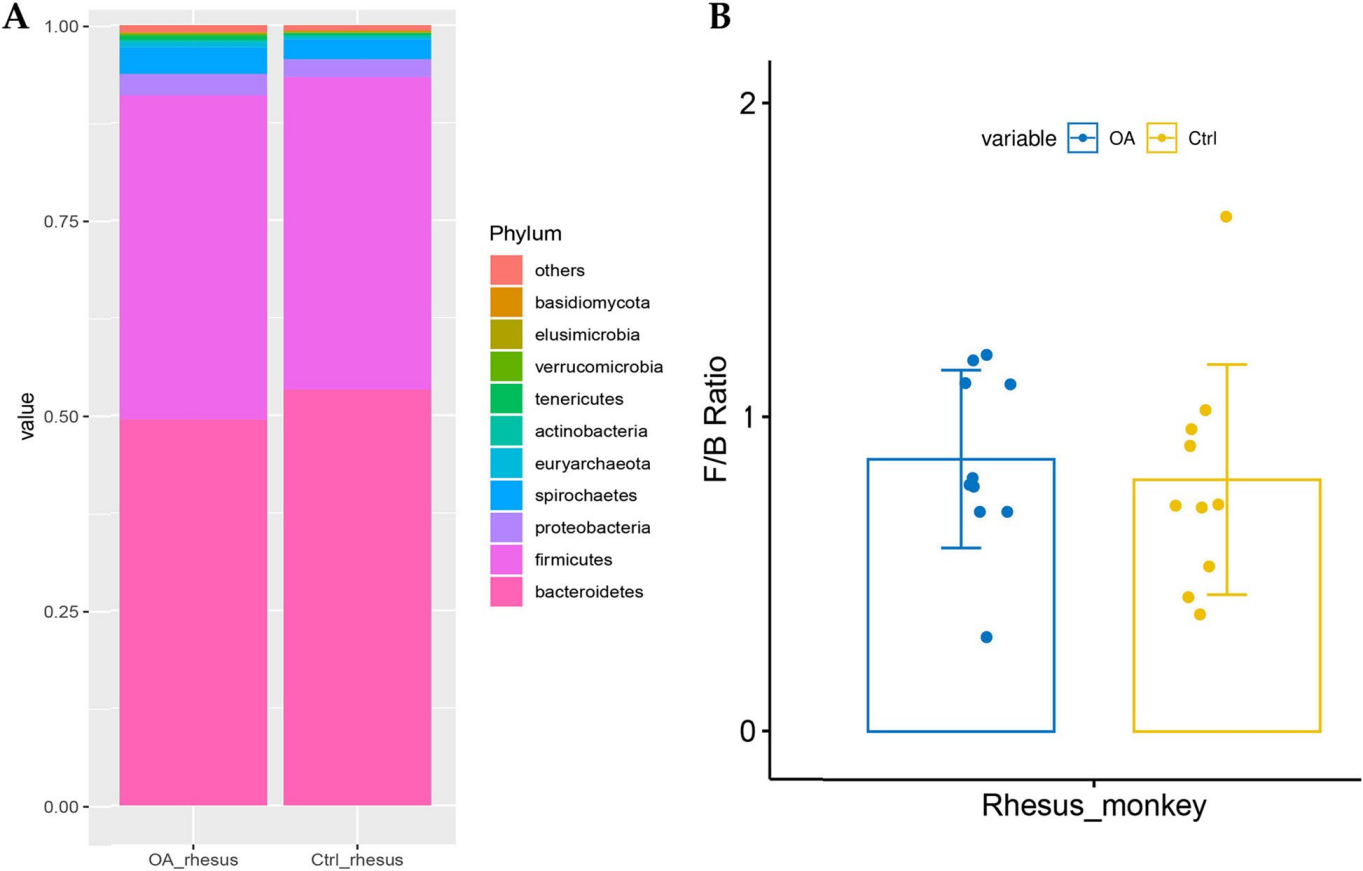
Analyze the microbiome composition at the phylum level. **A** The 10 most abundance phylum in the OA and normal monkeys. **B** The values of Firmicutes vs. Bacteroidetes of OA and normal monkeys were compared, but there was no significant difference

Bacteroidetes (> 50%) and Firmicutes (> 40%) both were the main phylum in the normal and OA monkeys (Fig. 3A). The value of Firmicutes vs. Bacteroidetes (F/B value) was increased in the OA group (*p* = 0.66) compared to normal monkeys (Fig. 3B).

**Fig. 4:**
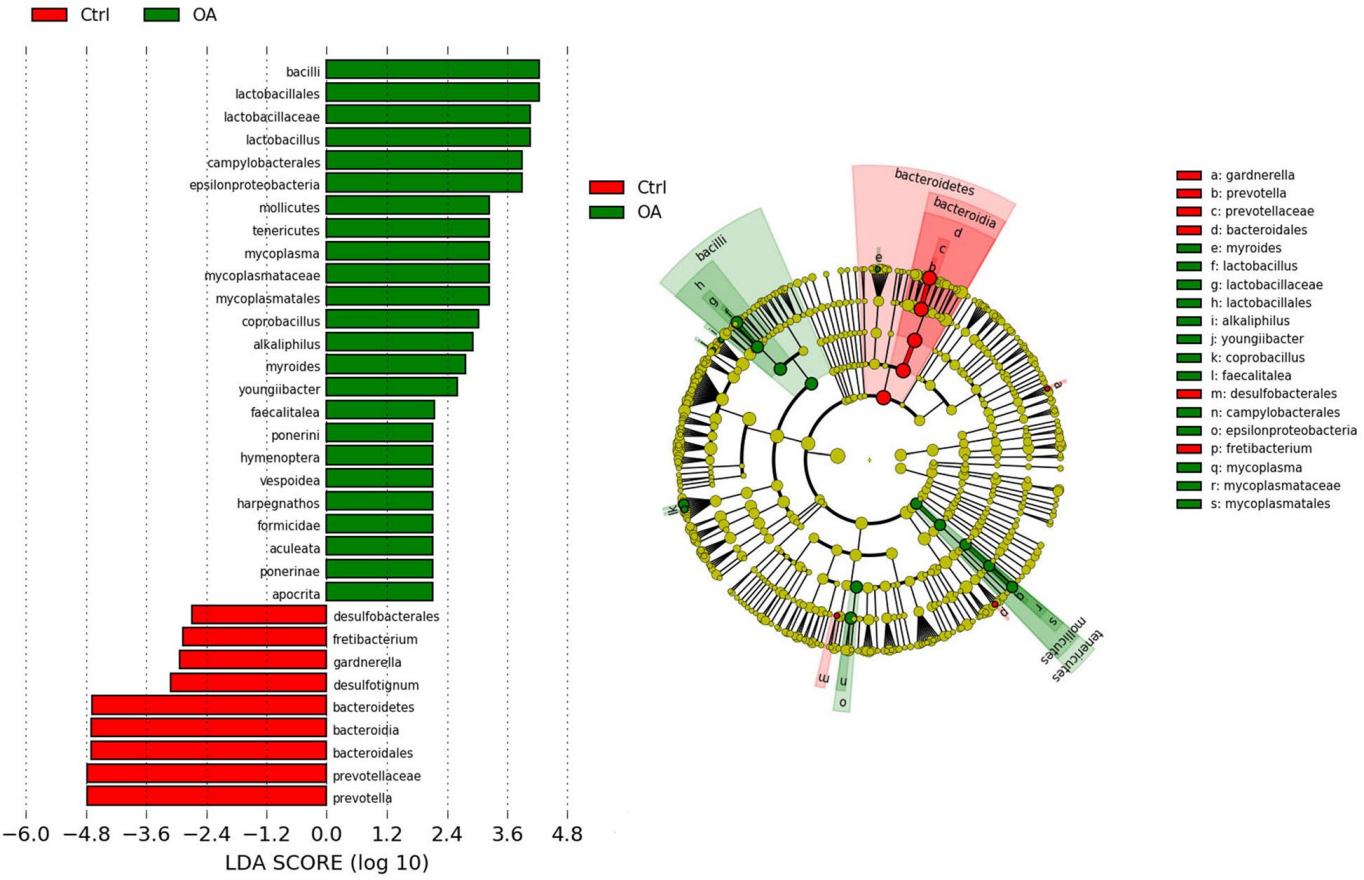
Taxonomic differences of gut microbiota in OA and normal monkey. **A** Linear discriminant analysis (LDA) and Linear discriminant analysis effect size (LEfSe) analysis revealed significant differences in gut microbiota between the OA (positive score) and normal groups (negative score). The LDA scores (log10) > 2 and *P* < 0.05 are listed. **B** Cladogram using LEfSe method indicating the phylogenetic distribution of gut microbiota associated with OA and control group

LDA and LEfSe analysis were used to compare the microbiota between OA and normal monkeys. Our results indicate that there is a significant difference in the gut microbiota between OA and normal monkeys based on LDA and LEfSe analysis. The relative abundances of the *Prevotella*, Prevotellaceae, Bacteroidales, Desulfobacterales, *Gardnerella* and *Fretibacterium* were higher in the normal group than OA group. Whereas, the relative abundances of *Lactobacillus*, Mollicutes, Tenericutes, *Coprobacillus* and *Faecalitalea* were higher in the OA monkeys compared to control group (Fig. 4A, B).

**Fig. 5:**
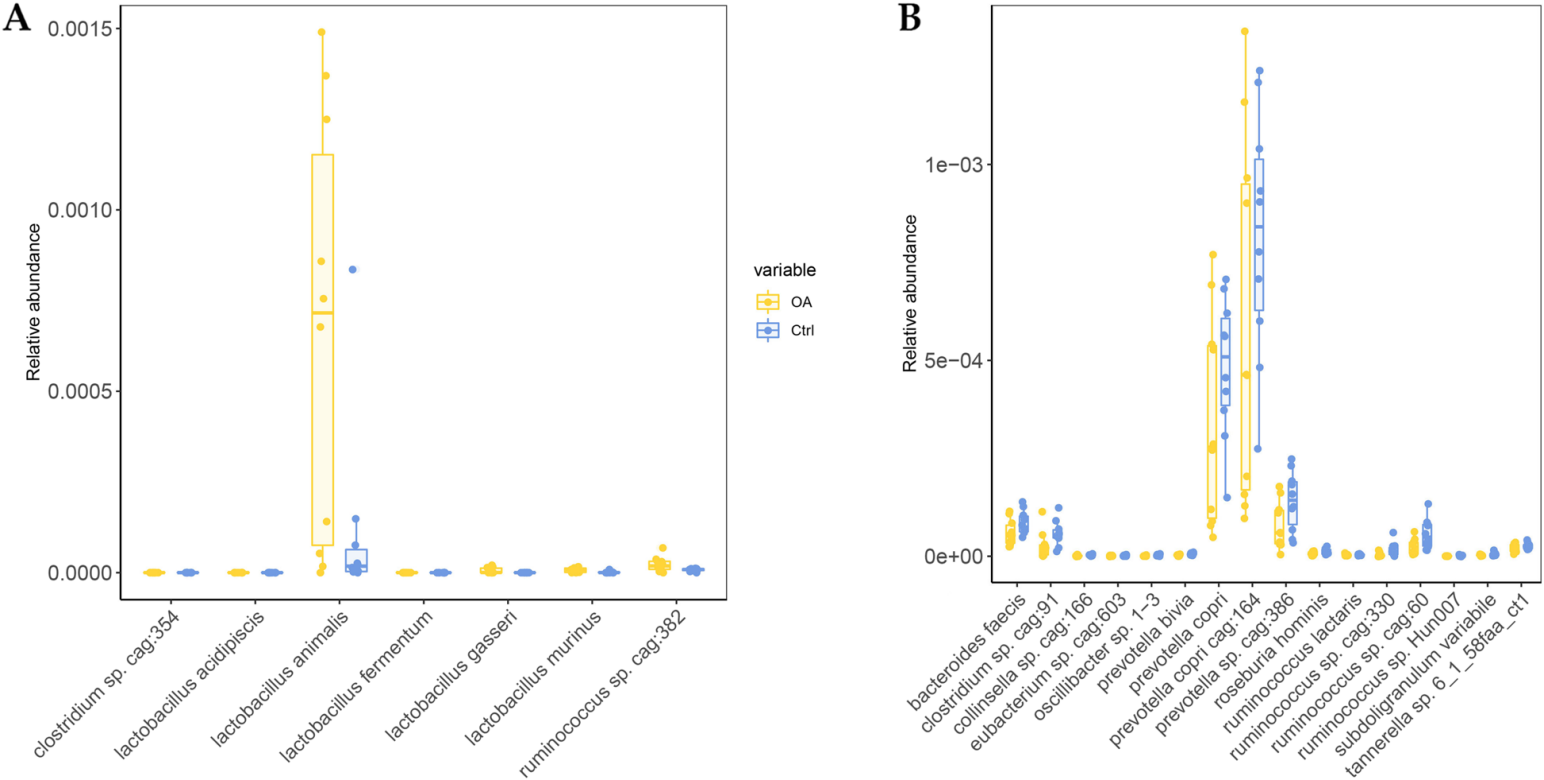
Analyze the composition of microbiota in species level. **A** The phylotypes significantly increased (*p* < 0.05) in the OA monkeys. **B** The phylotypes significantly decreased in the OA monkeys. Yellow and blue represent the OA group and normal groups respectively

At the species level, 23 species showed significant differences between the OA group and normal group. Seven species showed a higher prevalence in the OA group, and 16 species showed a higher prevalence in the normal group. *Lactobacillus acidipiscis* (*p* = 0.025), *Lactobacillus animalis (p* = 0.039), *Lactobacillus fermentum* (*p* = 0.039), *Lactobacillus gasseri (p* = 0.044), *Lactobacillus murinus* (*p* = 0.031) from *Lactobacillus* found significantly increased in OA monkeys (Fig. 5A). *Prevotella copri cag:164 (p* = 0.048), *Prevotella copri* (*p* = 0.038), *Prevotella sp. cag:386* (*p* = 0.011), *Prevotella biviawere* (*p* = 0.0056) from *Prevotella* significantly decreased in OA monkeys. *Ruminococcus sp. cag: 60* (*p* = 0.013), *Ruminococcus sp. cag: 330* (*p* = 0.048), *Ruminococcus lactaris* (*p* = 0.025), *Ruminococcus sp.* (*p* = 0.034) from *Ruminococcus* also were significantly decreased in OA monkeys (Fig. 5B).

### Predictive function analysis

**Fig. 6:**
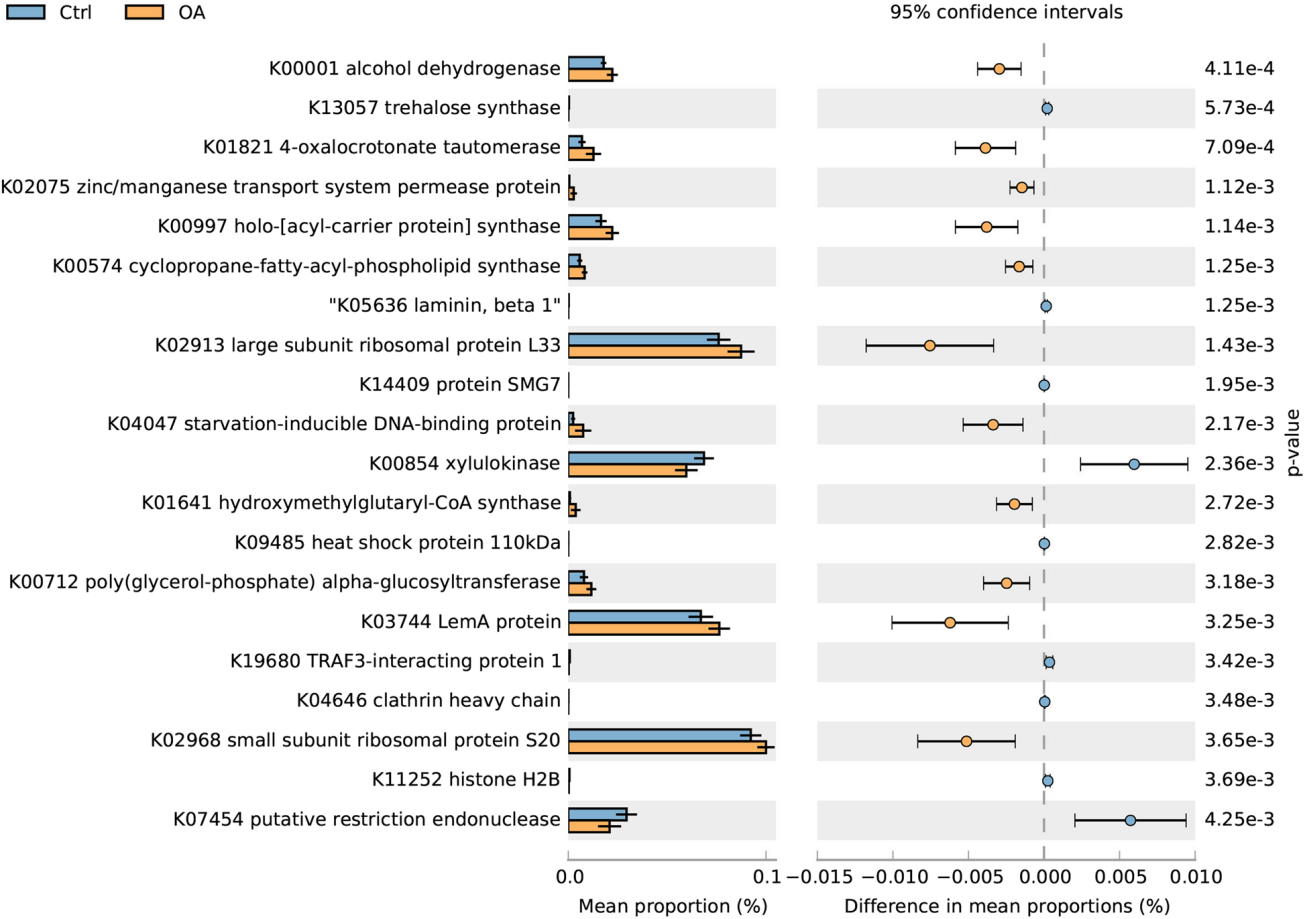
Analyze about functional predictions of gut microbiome. The KOs with significantly different abundances in the gut microbes identified using the software package PICRUSt (FDR, *P* < 0.05 are listed)

Phylogenetic investigation of Communities by Reconstruction of Unobserved States (PICRUSt) based on closed-reference operational taxonomic unit (OTU) was used to predict the abundances of functional categories the Kyoto Encyclopedia of Genes and Genomes (KEGG) ortholog (KO). A total of 20 KOs were identified with significantly different abundances in the gut microbes between the OA and normal monkeys (Fig. 6). Eleven pathways of function from gut microbiota were significantly enriched in the OA monkey, 9 pathways of function from gut microbiota were enriched in normal monkey. Zinc/manganese transport system permease protein (KO2075) (OR = 16, 95.0% lower CI = − 0.0023, 95.0% upper CI = − 0.00067, *p* = 0.0011) and Cyclopropane-fatty-acyl-phospholipid synthase (KO0574) were observed significantly increased in OA monkeys than normal monkeys (OR = 16, 95.0% lower CI = − 0.0025, 95.0% upper CI = − 0.00074, *p* = 0.0012).

## Discussion

Non-human primates are important animal models for drug development and safety evaluation in preclinical study. Rhesus monkey are similar to humans in walking style, upright body maintenance, and biomechanics [22]. The pathological changes and cartilage metabolism of spontaneous OA in rhesus monkeys are very similar to humans [18, 20, 21]. So rhesus monkeys with spontaneous osteoarthritis are the most suitable animal models for the study of OA. In the present study, we screened spontaneous OA monkeys in the colony by MRI and further analyzed the colonic contents of monkey with OA by metagenomic sequencing. We found that the composition and diversity of the gut microbes in monkeys with OA are different. The diversity of gut microbiota of OA monkeys decreased compared to the normal monkey but with no significant differences, which may be due to the fact that a limited number of monkeys were screened and involved in this study since the occurrences of spontaneous OA are rare in monkey. Previous studies have showed that alpha-diversity of microbiota from cartilage significantly decreased in OA patients compared to healthy people [15]. Furthermore, alpha-diversity of microbiota also was decreased in children with juvenile idiopathic arthritis compared to healthy children [23]. These results suggest that microbiota diversity has a downward trend both in OA patients and OA monkeys. In our study, the composition of the gut microbes has a separation in OA and normal monkeys by PCoA analysis, which indicates that the composition of gut microbes in OA and normal monkeys is very different.

Increases in the Firmicutes and Bacteroidetes (F/B) ratio in the gut have been linked to obesity [24]. Among our samples, F/B also has an increasing trend in OA monkeys. Prevotellaceae was significant enrich in normal monkeys, but significantly decreased in OA monkeys. On the contrary, Prevotellaceae was enriched in rheumatoid arthritis (RA) patients [25]. In addition, Bacteroidales was more enriched in healthy people compared to RA patients [25, 26]. Bacteroidales also was observed to been riched in normal monkeys compared to OA monkeys in our study. In OA patients, Bacilli, Lactobacillales, Lactobacillaceae, *Lactobacillus* and Mycoplasmataceae were more enriched in cartilage of knee compared to cartilage of hip [15, 27]. These microbes are also significantly enriched in the feces of OA monkeys compared to normal monkeys in our study. In genus level, *Prevotella* was decreased in children with juvenile idiopathic arthritis [23]. *Prevotella* was decreased in OA monkeys. *Prevotella copri* was significantly enriched in the intestines of RA patients [28]. In contrast, in our study, *Prevotella copri* was found significantly decreased in the feces of OA monkeys. *Ruminococcus* has been reported to be mainly enriched in RA patients [26], but in the present study, *Ruminococcus* significantly decreased in OA monkeys. The above results suggested that the composition of microbes has very large differences or even opposites exist between OA and RA. More importantly, the microbial from stool of monkey with spontaneous OA is very similar to the microbial composition of cartilage in patients with OA. Microbes with the consistent relative abundance in cartilage and feces may be the targets for diagnosis and intervention of OA.

Our functional analysis was performed using KEGG to identify microbiota functional pathways related to OA. Cartilage injury is the most important feature of OA. Cartilage is composed of extracellular matrix synthesized by chondrocytes, and the extracellular matrix is mainly composed of collagen and proteoglycan [29, 30]. Collagen is a protein that is synthesized in the ribosome. Large subunit ribosomal protein L33 (KO2913) and small subunit ribosomal protein S20 (KO2968) were significantly increased in OA monkeys indicating protein synthesis was increased, which may be related to cartilage injury. Keratan sulfate (KS) is also a component of cartilage. KS is synthesized by the glycosyltransferase in the golgi apparatus. With the increase of age, the content of KS also increases [30]. Poly (glycerol-phosphate) alpha-glucosyltransferase (KO0712) is a glycosyltransferase that is significantly elevated in OA monkeys, perhaps related to the synthesis of KS. Zinc/manganese transport system permease protein (KO2075) was observed significantly increased in OA monkeys. One of the detectable signs of cartilage damage is the increase of metalloproteinases. Metalloproteinases are a kind of extracellular matrix zinc protease [31], so KO2075 may be related to cartilage damage. Cyclopropane-fatty-acyl-phospholipid synthase (KO0574) also was enriched in OA monkeys, K00574 signaling is intimately linked with oxidative stress in OA [32].

Gut microbiome between captive and wild non-human primate showed that captivity “humanizes” the primate microbiome. Our results are largely consistent with the composition of microbiota in cartilage of OA patients. The monkeys participating in the experiment were kept in the same room in a single cage, and the diet remained consistent, we also ensured that the monkeys had not been exposed to antibiotics 3 months before sampling. Therefore, our research has evaded influence of these factors and ensured the results of the study will be more credible. However, the small number of animals and the fact that the research objectives only include feces without involving the colonic mucosa also limit overall research on the intestinal microbiota.

## Conclusions

Nonhuman primate models can well simulate the occurrence and development of human diseases. Our results indicate that the diversity and composition of intestinal microbiota in monkeys with OA are different compared to the normal monkeys. Mollicutes, Tenericutes, *Coprobacillus* and *Faecalitalea* may be biomarkers for the monkeys of OA. *Prevotella* and Desulfobacterales were higher in the normal group than OA group. The functional analysis of the microbiota also predicts cartilage damage. Our results are largely consistent with the composition of microbiota in cartilage of OA patients. This study may provide a very valuable reference for the future development of microbial preparations for the treatment of osteoarthritis.

## Methods

### Animals

Twenty adult rhesus macaques (female) at the ages of 6–15 years old were provided by State Key Laboratory of Primate Biomedical Research of Kunming University of Science and Technology. Adult rhesus macaques at were individually caged, which included ten macaques diagnosed as spontaneous osteoarthritis and ten health rhesus macaque as normal control, which included ten macaques diagnosed as spontaneous osteoarthritis and ten health rhesus macaque as normal control. Ten monkeys with osteoarthritis were screened from the monkey population. These monkeys may have maintained osteoarthritis symptoms for at least 2 years. All of the animals were maintained in a 12 h light: 12 h darkness cycle, temperature was kept at 18–26 °C and humidity from 40 to 70%. All procedures were approved by the Institutional Animal Care and Use Committee of Kunming University of Science and Technology (protocol number: LPBR20170201), and were carried out in accordance with the Guide for the Care and Use of Laboratory Animals (8th edition).

### Identification of spontaneous osteoarthritis in rhesus macaques

The animals were anesthetized by intramuscular injection of ketamine with a volume of 5 mg/kg. The magnetic resonance imaging (MRI) scan was conducted on the 3 T machine (Siemens). The MRI scanning of the knees was performed on the rhesus monkeys. Articular cartilage was quantitatively assessed based on T1 rho (TR: 420.0 TE: 12.0) and T2 (TR: 3090.0 TE: 12.0) relaxation times. Cartilage thickness and signal intensity of the surfaces of the patella, medial and lateral femoral were measured.

### Fecal sample collection and DNA extraction

Fresh fecal samples were collected in sterile tubes from the 20 rhesus macaques. Then, the fecal samples were transferred to the laboratory immediately in an ice bath and stored at − 80 °C (not more than 3 months). The isolation of purified microbial genomic DNA was performed from each fecal sample using a MoBioPowerSoil® DNA Extraction Kit (Arlsbad, CA, USA) according to the manufacturer’s recommendation. The DNA concentration was measured using Qubit® DNA Assay Kit in Qubit® 2.0 Flurometer (Life Technologies, CA, USA).

### Library preparation for sequencing

Each sample needed a total amount of 700 ng DNA to be used as input material for the DNA sample preparations. According to the manufacturer’s recommendation, sequencing libraries were generated using NEB Next® Ultra DNA Library Prep Kit for Illumina® (NEB, USA), and index codes were added to attribute sequences for each sample.

### Clustering and sequencing

In the cBot Cluster Generation System, the clustering of the index-coded samples was performed by HiSeq 4000 PE Cluster Kit (Illumina) according to the manufacturer’s instructions. After cluster generation, the library preparations were sequenced on an Illumina Hiseq 4000 platform and 150 bp paired-end reads were generated.

### Metagenome data analyses

#### Assembly of the metagenome and construction of the gene catalog

Raw paired-end reads were processed to exclude: (1) adaptor sequences; (2) low-quality reads that have more than 40% of bases with a quality score < 5, (3) reads containing more than 10% unknown bases; (4) reads mapped to host genome (NCBI Reference genome: Mmul_8.0.1/rheMac8, *Macaca mulatta*) by BWA-MEM [33]. Finally, paired reads longer than 75 bp were selected as high-quality-reads. For each sample, Megahit v1.0.6 [34] was used to assemble the high-quality-reads under pair-end mode with default parameters, respectively. Prodigal v2.6.3 [35] was used to perform gene prediction using contigs (a length threshold of 500 bp) with parameter “-p meta”. Then, the non-redundant gene catalog was constructed using cd-hit-est v4.6.6 [36] based on the predicted ORFs (length longer than 100 bp were selected), and the redundant genes were removed using a sequence identity cut-off of 0.95. Additionally, the functional assignments of the non-redundant proteins were performed based on the Kyoto Encyclopedia of Genes and Genomes (KEGG) database.

#### Taxonomic annotation

Taxonomic annotation of protein sequences generated by Prodigal were performed by DIAMOND v0.8.28.90 [37] alignment against the NCBI-NR database using CARMA3 [38] with the default parameters. To obtain the relative gene abundance, the high-quality-reads from each sample were aligned against the non-redundant gene catalog by BWA-MEM using the criteria of length ≥ 50 bp and identity > 0.95. The sequence-based relative abundance calculation referred to a previously described method. The relative abundances of phylum, genus, species and KO were calculated by summing the abundance of genes belonging to each category for each sample. Metastats analysis was conducted to investigate the difference of the relative abundance for each species and gene between the two groups [39]. A multi-comparison adjusted Q < 0.05 was used to define significant differences.

#### Microbial composition analysis

For microbial diversity analysis, Shannon index and Simpson index were used to describe the α-diversity (intergroup diversity), using R package “vegan”. A PCoA analysis were performed to describe the β-diversity (intragroup diversity) by the R package “vegan” (vegdist was used to calculate the Bray-Curtis dissimilarity values), ggplot2 was used to do visualization. The difference tests of alpha diversity for different groups were performed using Wilcoxon Rank Sum Test. Beta diversity on unweighted UniFrac were calculated by QIIME software (v1.7.0).

#### Discovery of biomarkers

The genomic features (organisms and clades) were identified by a metagenomic biomarker discovery approach called Linear discriminant analysis Effect Size (LEfSe: https://huttenhower.sph.harvard.edu/galaxy/) [40]. Kruskal-Wallis and pairwise Wilcoxon tests were implemented, followed by a Linear discriminant analysis (LDA) to evaluate the effect size determined by LEfSe of each differentially abundant taxon. Bacteria with considerably increased values were defined as those with an LDA score (log10) of over 2. By way of class comparison, tests of biological consistency and effect size estimation to address the differences between multi microbial communities.

## Data Availability

The obtained metagenomic profiles have been uploaded into the NCBI SRA database and are accessible via the accession number: PRJNA732758.

## Abbreviations

OA: Osteoarthritis
LPS: Lipopolysaccharide
LDA: Linear discriminant analysis
LEfSe: Linear discriminant analysis effect size
OUT: Operational taxonomic unit
KEGG: Kyoto Encyclopedia of Genes and Genomes
KO: Ortholog
RA: Rheumatoid arthritis
MMP-1: Matrix metallopeptidase 1
